# LncRNA SNHG14/miR-5590-3p/ZEB1 positive feedback loop promoted diffuse large B cell lymphoma progression and immune evasion through regulating PD-1/PD-L1 checkpoint

**DOI:** 10.1038/s41419-019-1886-5

**Published:** 2019-09-30

**Authors:** Lina Zhao, Ye Liu, Jingbo Zhang, Yan Liu, Qi Qi

**Affiliations:** 1 Department of Hematology, The Tumor Hospital Affiliated to Harbin Medical University, Harbin, Heilongjiang China; 2 Department of Immunology Teaching and Research, Harbin Medical University, Harbin, China

**Keywords:** Cancer, Cell biology

## Abstract

Diffuse large B cell lymphoma (DLBCL) is the commonest disorder derived from the B-lymphocytes. Inhibiting the immune checkpoint through naturalizing programmed death-1 (PD-1) and programmed death ligand 1 (PD-L1) is proved to be a successful therapeutic regime for lymphoma. Long non-coding RNAs (lncRNAs) are unceasingly reported to be promising biological targets for the cancer therapies. This study planned to explore the regulation of small nucleolar RNA host gene 14 (SNHG14) on DLBCL. SNHG14 level in DLBCL samples and cell lines was analyzed by GEPIA bioinformatics tool and RT-qPCR. Biological functions of SNHG14 in DLBCL were detected by CCK-8, colony formation, and transwell invasion assays. Molecular interaction was determined by RNA immunoprecipitation (RIP) and luciferase reporter assays. MiR-5590-3p-related pathway was identified through KEGG pathway analysis applying DAVID6.8 online bioinformatics tool. Effect of SNHG14 on CD8+ T cells was detected by flow cytometry. Results depicted that SNHG14 was upregulated in DLBCL and its depletion retarded proliferation, migration and epithelial-to-mesenchymal transition (EMT). Mechanistically, SNHG14 sponged miR-5590-3p to upregulate Zinc finger E-box binding homeobox 1 (ZEB1), and ZEB1 transcriptionally activated SNHG14 and PD-L1 to promote the immune evasion of DLBCL cells. In conclusion, we firstly showed that SNHG14/miR-5590-3p/ZEB1 positive feedback loop promoted diffuse large B cell lymphoma progression and immune evasion through regulating PD-1/PD-L1 checkpoint, indicating that targeting SNHG14 was a potential approach to improve the efficacy of immunotherapy in DLBCL.

## Introduction

Diffuse large B cell lymphoma (DLBCL) is the most prevalent subtype of non-Hodgkin lymphoma (NHL), accounting for over one-third of all NHL cases globally^1,2^. DLBCL is a highly aggressive hyperplastic disorder in lymphatic system, and there are around 40% of DLBCL patients presenting resistance to the clinical therapeutic tools available currently^3^.

Of note, researches have provided compelling evidence that immune evasion is essential to tumor survival and development^4,5^. It has been demonstrated that in tumor microenvironment, tumor cells recruit immunosuppressive cells, such as CD4+ T cells, to disturb the cytotoxic functions of CD8+ T cells^6–8^. Furthermore, programmed death ligand 1 (PD-L1), a B7 family ligand, can interact with its receptor programmed death-1 (PD-1) to modulate tumor-specific T cells^9,10^. PD-L1 restrains the activity and induces the apoptosis of CD8+ T cells through binding to PD-1, leading to the immune evasion in tumors^11–13^. Blocking PD-1/PD-L1 checkpoint by antibodies against PD-L1 or PD-1 is considered as an efficient tool for the immunotherapy in tumors^14–17^, including lymphoma^18,19^. Hence, identification of biological targets associated with the regulation of PD-1/PD-L1 in DLBCL is helpful to improve the clinical efficacy of immunotherapy in DLBCL.

Zinc finger E-box binding homeobox 1 (ZEB1) is a transcriptional factor (TF) demonstrated as an oncogene regulating invasion, migration, EMT, and proliferation of diverse types of cancer cells^20–22^. As a TF, ZEB1 could either activate or repress the transcription of target genes via recruiting different cofactors^23–26^. Moreover, reports have shown that ZEB1 upregulation contributes to the aggressive behavior of diffuse large B cell lymphoma^27^. Interestingly, recent studies have shown that ZEB1 could upregulate the expression of PD-L1 to improve the immune evasion in cancer cells^28,29^. However, the detailed mechanism behind the regulation of ZEB1 on PD-L1 in DLBCL needs to be further elucidated.

Long non-coding RNAs (lncRNAs) are a series of non-coding RNAs comprised of more than 200 nucleotides^30^. Although lncRNAs possess little protein-coding capacity, they are suggested as promising markers for the prognosis, diagnosis, and development of cancers^31–33^. The association of lncRNAs with carcinogenesis in myriads types of cancers has been well established^34,35^, including with DLBCL^36,37^. However, the impact of lncRNAs on PD-1/PD-L1 pathway and immune evasion in DLBCL is rarely explored. Small nucleolar RNA host gene 14 (SNHG14) is documented to elicit oncogenic functions by modulating proliferation, migration, invasion, and conferring chemo-resistance in multiple types of malignancies, such as gastric cancer, clear cell renal cell carcinoma, and breast cancer^38–40^. Nevertheless, SNHG14 has never been related to DLBCL and PD-1/PD-L1 immune checkpoint before.

MicroRNAs (miRNAs) are recognized as short non-coding RNAs that consist of 22 nucleotides^41^. They are commonly characterized as repressors of gene expression since through base-pairing, miRNAs can target the 3’ untranslated region (3’UTR) of mRNAs and result in the degradation of mRNAs and decrease of protein production^42,43^. LncRNAs are largely reported to function through competitive endogenous RNA (ceRNA) mechanism, whereby lncRNAs interact with miRNAs to prevent miRNAs from targeting and inhibiting downstream genes^44,45^. To date, miRNAs are shown to regulate a wide range of cellular activities related to cancer development, including the escape from the anti-tumor immune response^28,46^. MiR-5590-3p has been previously investigated in gastric cancer and breast cancer, and is indicated as a tumor-suppressive gene which prevents cell proliferation and migration^47,48^. However, miR-5590-3p has neither been investigated in DLBCL nor been related to SNHG14, ZEB1, and PD-1/PD-L1.

This paper aimed to investigate the modulation of SNHG14 on DLBCL progression and PD-1/PD-L1-mediated immune evasion.

## Materials and methods

### Tissues

Thirty-eight paired B cell lymphoma tissues and adjacent normal tissues were obtained from the Tumor Hospital Affiliated to Harbin Medical University. This study was carried out with the approval of the ethics committee of the Tumor Hospital Affiliated to Harbin Medical University. All participants had signed informed consent. Tissue samples were frozen at −80 °C the moment they were attained. No patients had received chemotherapy or radiotherapy before surgery.

### Microarray assay

Three pairs of DLBCL tissues and adjacent normal tissues were applied for lncRNA or miRNA microarray analysis. Differentially expressed lncRNAs or miRNAs were analyzed using GeneChip Operating Software (Affymetrix, Santa Clara, CA, USA). Fold Change > 2.0 and *P* < 0.05 were the criteria of selecting lncRNAs or miRNAs for follow-up analyses.

### Cell culture

Human lymphoblastoid B cell (GM12878), human renal epithelial cell (293T), murine DLBCL cell (A20), and human DLBCL cells (OCI-LY7, DB, U2932, and FARAGE) were purchased from American Type Culture Collection (ATCC; Manassas, VA, USA). GM12878 cells were grown in RPMI 1640 (HyClone, Logan, UT, USA) with GlutaMAX Supplement (Thermo Fisher Scientific, Waltham, MA, USA), 15% fetal bovine serum (FBS; Sigma-Aldrich, St. Louis, MO, USA), and 1% pen/strep (Invitrogen, Carlsbad, CA, USA). DLBCL cells were grown in RPMI 1640 with 10% FBS and 0.05 mg/mL gentamycin (Schering-Plough Europe, Brussels, Belgium). 293T cells were grown in DMEM (Thermo Fisher Scientific) containing 10% FBS and 1% pen/strep. Cells were cultured at 37 °C in 5% CO_2_.

### Cell transfection

Specific shRNAs against SNHG14 (sh-SNHG14#1/2/3) or ZEB1 (sh-ZEB1#1/2/3), sh-NC, pcDNA3.1 vector targeting SNHG14 or ZEB1, and the empty vector were constructed by Genechem (Shanghai, China). MiR-5590-3p mimic/inhibitor and NC mimic/inhibitor were designed by GenePharma (Shanghai, China). These plasmids were transfected into FARAGE or U2932 cells as required via Lipotransfectamine 3000 (Thermo Fisher Scientific).

### Quantitative real-time PCR (RT-qPCR)

Extraction of total RNA applying TRIzol reagent (Invitrogen) was performed before synthesis of cDNA via a cDNA synthesis kit (Thermo Fisher Scientific). Relative expression levels were detected by RT-qPCR with SYBR Premix Ex Taq II (Takara, Tokyo, Japan) and an ABI7000 sequence detector (Applied Biosystems, Foster City, CA, USA) and calculated by 2^−∆∆Ct^ method. Glyeraldehyde-3-phosphate dehydrogenase (GAPDH) or U6 was normalization.

### CCK-8 and colony formation assays

Transfected FARAGE or U2932 cells seeded in 96-well plates (1 × 10^3^ cells/well) were incubated for 0, 24, 48, 72, and 96 h. Thereafter, the cells were subjected to the addition of cell counting kit 8 (CCK-8; Dojindo, Tokyo, Japan) and incubated for 2 h. The optical density was measured at 450 nm.

For colony formation assay, transfected cells in 6-well plates (5 × 10^2^ cells/well) were cultured for two weeks. Then, the cells were subjected to the fixation using methanol and then stained using crystal violet (Sigma-Aldrich). The number of colonies containing more than 50 cells was counted manually.

### Transwell invasion assay

24-well transwell chambers (BD Biosciences, Franklin Lakes, NJ, USA) were used for cell invasion. FARAGE or U2932 cells in serum-free medium were seeded into upper chambers pre-coated with 2% Matrigel (BD Biosciences), and medium containing 20% FBS was added to the lower chambers. After being incubated for 48 h, the non-invaded cells were scrapped off using a cotton swab, and the cells invaded through the matrigel underwent fixation and were stained by with crystal violet. Five fields were randomly selected in each well and the invaded cells were observed and counted under an IX71 microscope (Olympus, Japan) with Image-Pro Insight software (Olympus, Japan).

### Western blotting

Proteins were extracted from transfected FARAGE or U2932 cells using RIPA lysis buffer (Beyotime, Shanghai, China) and loaded, run on 10% SDS-PAGE (Bio-Rad, Hercules, CA, USA), followed by the transferring to PVDF membranes (Millipore, Bedford, MA, USA). After being blocked with 5% nonfat milk, membranes were incubated with primary antibodies including E-cadherin (ab194982, Abcam, Cambridge, USA), N-cadherin (ab202030, Abcam), ZEB1 (ab203829, Abcam), PD-L1 (ab205921, Abcam) and GAPDH (ab8245, Abcam), followed by 1 h of incubation with secondary antibodies, and detected by ECL reagents (Pierce, Rockford, IL, USA).

### Immunofluorescence (IF)

IF was carried out in line with previous description^49^. In short, DLBCL cells with indicated transfection were fixed in 4% paraformaldehyde and underwent permeabilization in 0.2% Triton X-100 (Sigma-Aldrich). After the blocking in PBS with 10% goat serum, cells were incubated with the primary antibodies against E-cadherin and N-cadherin (Abcam, Cambridge, UK) overnight at 4 °C, followed by 1 h incubation with appropriate rhodamine-conjugated secondary antibodies. Then, cells were washed and subjected to nuclear staining with DAPI (Invitrogen). A laser scanning Olympus microscope was applied to monitor the immunofluorescence.

### Luciferase reporter assay

The pmirGLO dual-luciferase vector (Promega, Madison, WI, USA) containing SNHG14 sequence was co-transfected with miRNA mimic into 293T cells. The SNHG14-WT/Mut or ZEB1-WT/Mut was co-transfected with miR-5590-3p mimic or NC mimic into 293T cells. PD-L1 promoter WT/Mut or SNHG14 promoter WT/Mut was sub-cloned into the pGL3-basic vector (Promega), then co-transfected into 293T cells with sh-ZEB1 or sh-NC. Luciferase activities were explored via Dual-Luciferase Reporter Assay System (Promega).

### RNA immunoprecipitation (RIP)

RIP was implemented by a Magna RIP™ RNA-Binding Protein Immunoprecipitation Kit (Millipore). Cells with indicated transfection were harvested and then lysed in the lysis buffer (50 mM Tris−HCl, pH = 7.4, 150 mM NaCl, 1% Triton-100, 0.1% SDS, 1.5 mM EDTA). Thereafter, cell lysates underwent incubation with RIP buffer which contained magnetic beads. The beads were conjugated with Argonaute 2 antibody (Abcam, Cambridge, UK) or anti-IgG (Abcam) as negative control. Then, the samples were digested applying Dnase I and Proteinase K, followed by the isolation of immunoprecipitated RNA. Eventually, the enrichment of purified RNAs was detected by RT-qPCR.

### Flow cytometry

A double-chamber co-cultured system (Millipore) was employed for co-culture. The Transfected FARAGE or U2932 cells were seeded in the upper chamber whereas the CD8+ T cells were in the lower chamber, allowing the direct contact of DLBCL cells with immune cells. Anti PD-1 (Abcam) or anti-PD-L1 (Abcam) was added. CD8+ T cells were sorted by using a EasySep™ Human CD8+ T Cell Isolation Kit (STEMCELL,Vancouver, BC, Canada). The percentage of CD8+ T cells or CD8+ T cell apoptosis was detected by flow cytometry with the application of Annexin V-FITC Apoptosis Kit (Becton Dickinson, Franklin Lakes, NJ, USA) in reference to the manufacturer’s instructions^50,51^.

### Chromatin immunoprecipitation (ChIP)

ChIP was performed by the application of SimpleChIP® Enzymatic Chromatin IP Kit (Magnetic Beads) #9003 (Cell Signaling Technology, USA) in accordance with the manufacturer's instructions as per previous description^52^. Antibodies against ZEB1 was applied to mmuno-precipitate the crosslinked protein-DNA complex, with anti-IgG as negative control. The immunoprecipitated DNA underwent purification and was analyzed by RT-qPCR with primers specific for the predicted binding sites on the promoter of SNHG14 or PD-L1.

### Nude mouse xenograft model

BALB/c mice (4-week-old) from Shanghai Laboratory Animal Center (Shanghai, China) were injected with transfected A20 cells (1 × 10^7^) into the right flank. Anti-PD-L1 antibody (200 μg/mouse) was injected into mice thrice each week for two weeks. Tumor volume was calculated every 4 days. Tumor weight was measured after mice were killed. Calculation of tumor volume was based on 0.5 × a (length) × b (width)^2^.

### Hematoxylin-eosin (HE) staining

To examine the metastasis in vivo, transfected A20 cells were injected into mice from tail vein and the mice were treated with or without anti-PD-L1. Tumor tissues were extracted from xenograft tumors for HE staining based on previous description^53^. In short, samples of xenografts embedded in paraffin were sectioned to the 3 μm thickness and underwent HE staining. Each section was observed by a LSM 710 META confocal laser scanning microscope (Zeiss, Shanghai, China).

### Statistical analysis

Data were presented as mean ± SD. Variance analyses were implemented via Student's *t* test or one-way ANOVA. Pearson Correlation Coefficient was utilized for verifying significance of the correlation among SNHG14, miR-5590-3p and ZEB1 expression. *P* < 0.05 was considered statistically significant. Statistical analyses were conducted employing SPSS 22.0 (IBM, Armonk, NY, USA). All assays were implemented thrice.

## Results

### SNHG14 was upregulated in DLBCL, and promoted proliferation, invasion, and EMT

**Fig. 1:**
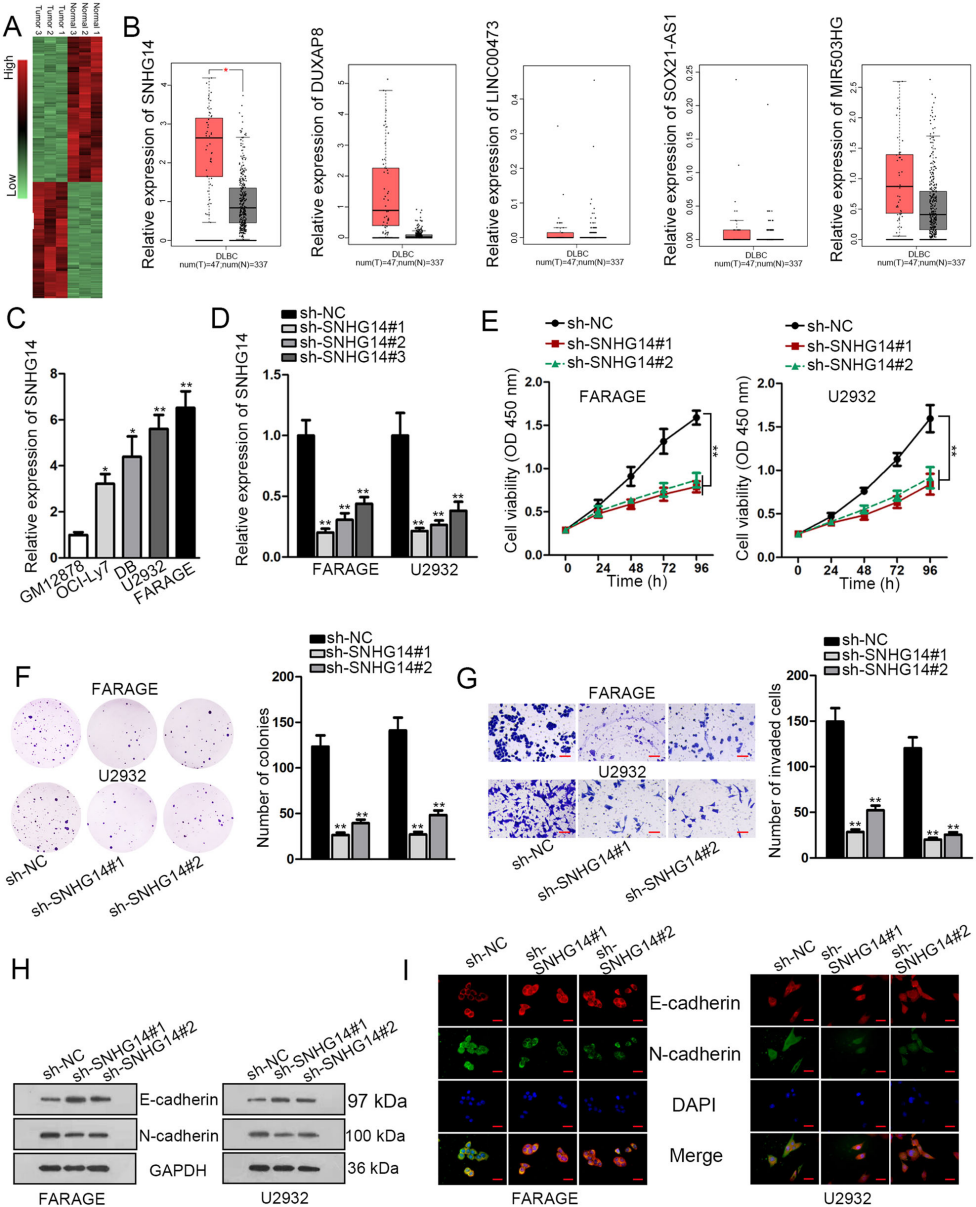
Expression and biological function of SNHG14 in DLBCL. **a** Hierarchical clustering showed the differentially expressed lncRNAs in DLBCL tissues compared with the paired para-tumor tissues according to the microarray analysis (Fold change > 2, *P* < 0.05). **b** The expressions of top-5 upregulated lncRNAs in DLBCL tissues in TCGA DLBCL samples were analyzed through GEPIA. **c** RT-qPCR data showed the upregulated expression of SNHG14 in DLBCL cell lines. **d** Knockdown of SNHG14 in FARAGE and U2932 cells was confirmed by RT-qPCR. **e**–**f** Viability and colony generation of DLBCL cells were evaluated by CCK-8 and colony formation assays. **g** Invasion of DLBCL cells was detected by transwell invasion assay. Scale bar: 100 μm. **h**–**i** EMT markers (E-cadherin and N-cadherin) were detected by western blot and IF staining assay in DLBCL cells. Scale bar: 50 μm. *P < 0.05, **P < 0.01

First, we applied microarray analysis to detect the differentially expressed lncRNAs in DLBCL in 3 pairs of DLBCL specimens and the matched adjacent non-tumor specimens. Consequently, we picked 5 lncRNAs that presented the most significant upregulation in DLBCL samples, which were SNHG14, DUXAP8, LINC00473, SOX21-AS1, and MIR503HG (Fig. 1a). By analyzing TCGA data through GEPIA (http://gepia.cancer-pku.cn/), we found that among the 5 lncRNAs, only SNHG14 exhibited significant high expression in DLBCL samples (Fig. 1b), further indicating the association of SNHG14 with DLBCL. Accordingly, high expression of SNHG14 was confirmed in DLBCL cell lines versus the normal B cell lymphocytes (Fig. 1c).

Later on, biological effect of SNHG14 in DLBCL was detected through in vitro loss-of-function assays. Two DLBCL cell lines, FARAGE and U2932, were applied in the experiments because they were verified to express the highest SNHG14 level among 4 DLBCL cell lines. RT-qPCR analysis confirmed the pronounced downregulation of SNHG14 in both DLBCL cell lines after the transfection of 3 SNHG14 specific shRNAs, and sh-SNHG14#1/2 silenced SNHG14 expression more significantly (Fig. 1d). Therefore, sh-SNHG14#1/2 were used for subsequent experiments. Depletion of SNHG14 impaired the viability and colony generation of two DLBCL cell lines (Fig. 1e, f). Invasive ability of DLBCL cells was weakened by SNHG14 knockdown (Fig. 1g). In addition, we tried to examine the EMT progression of DLBCL cells under SNHG14 silence. Western blot and IF staining results depicted that E-cadherin was increased, whereas N-cadherin was decreased by the knockdown of SNHG14 in DLBCL cells (Fig. 1h, i). Together, it was suggested that SNHG14 was upregulated in DLBCL and served as an oncogene by promoting cell proliferation, invasion, and EMT.

### SNHG14 interacted with miR-5590-3p in DLBCL cells

**Fig. 2:**
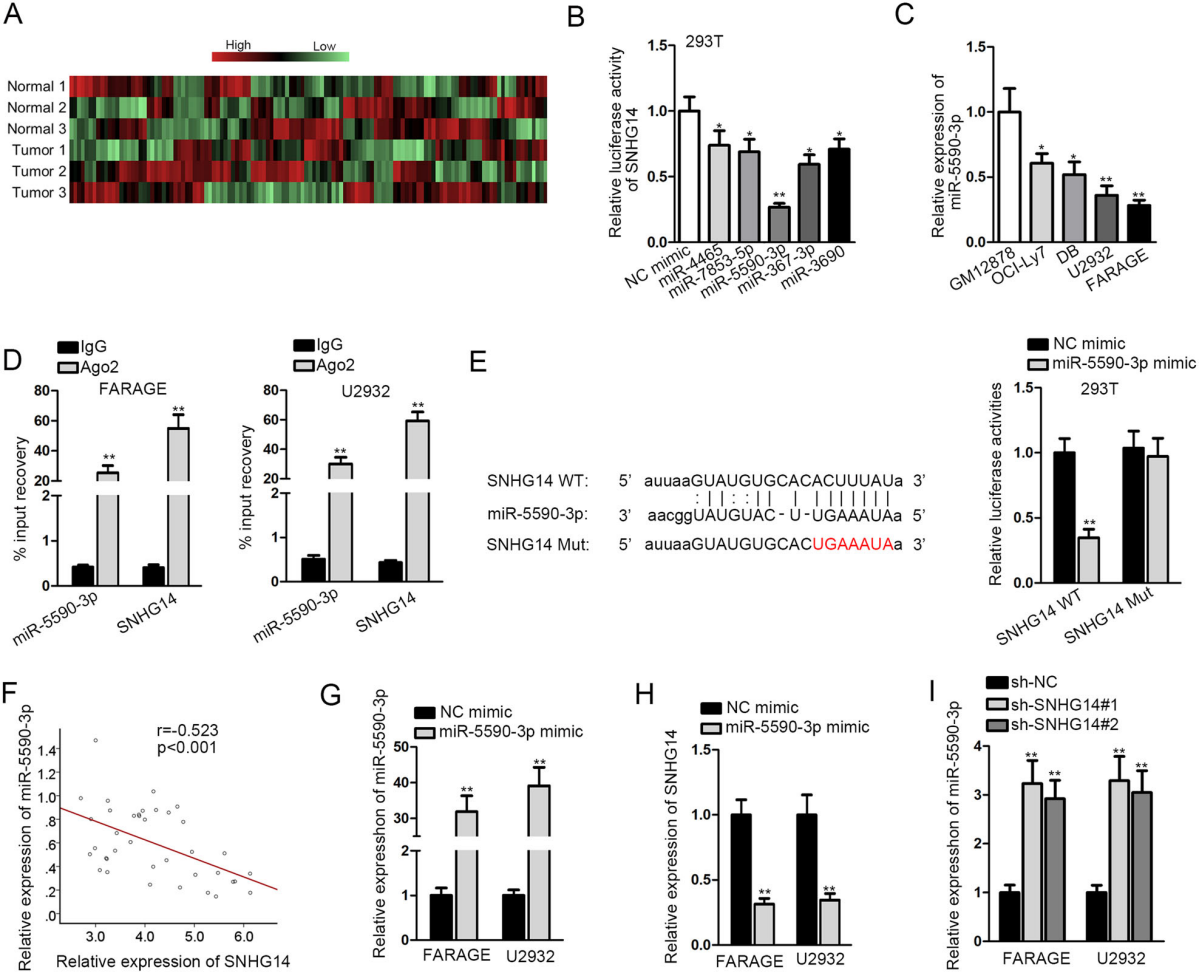
Interaction between SNHG14 and miR-5590-3p in DLBCL. **a** Heat map showed the expressions of 124 miRNAs potentially targeted by SNHG14 in 3 DLBCL tissues compared with the matched non-tumor tissues. **b** Luciferase reporter assay was conducted to detect the interaction between the top 5 downregulated miRNAs with SNHG14. **c** RT-qPCR analysis of miR-5590-3p expression in DLBCL cell lines and normal cell line. **d** RT-qPCR analysis following the RIP assay was conducted to confirm the interaction between miR-5590-3p with SNHG14 in DLBCL cells. **e** Interacting sequences on SNHG14 for miR-5590-3p were obtained from Starbase3.0 and mutated by altering them with the complementary sequences. Luciferase reporter assay was performed to detect the interaction between SNHG14 with miR-5590-3p. **f** Pearson’s correlation curve showed the negative relation between SNHG14 and miR-5590-3p in DLBCL tissues. **g** Overexpression of miR-5590-3p in DLBCL cells was confirmed by RT-qPCR assay. **h** Expression of SNHG14 upon miR-5590-3p overexpression in DLBCL cells was detected by RT-qPCR. **i** Expression of miR-5590-3p upon SNHG14 silence in DLBCL was detected by RT-qPCR. *P < 0.05, **P < 0.01

In subsequence, we detected the mechanism of SNHG14 in DLBCL. Large volumes of studies have elucidated the role of lncRNAs as miRNA sponges in cancer development^44,45^. Also, SNHG14 has been demonstrated to interact with several miRNAs such as miR-145, and miR-206-3p^38,54^. Therefore, we tried to investigate whether SNHG14 interacted with miRNA to regulate DLBCL. The prediction results of Starbase3.0 (http://starbase.sysu.edu.cn/) showed that 124 miRNAs putatively interacted with SNHG14. RT-qPCR analysis revealed that among 124 miRNAs, the 5 most downregulated miRNAs in DLBCL samples compared to the paired normal samples were miR-4465, miR-7853-5p, miR-5590-3p, miR-367-3p, and miR-3690 (Fig. 2a), indicating the association of these miRNAs with DLBCL. Luciferase reporter assay showed that among the 5 abovementioned miRNAs, miR-5590-3p overexpression led to the most significant reduction of luciferase activity of SNHG14 reporter (Fig. 2b), which suggested that miR-5590-3p presented the strongest association with SNHG14. Thus, we focused on exploring the interaction between SNHG14 and miR-5590-3p. Low expression of miR-5590-3p was validated by RT-qPCR analysis (Fig. 2c). The co-immunoprecipitation of miR-5590-3p and SNHG14 in DLBCL cells indicated that miR-5590-3p and SNHG14 potentially interacted in a RNA-induced silencing complex (RISC) (Fig. 2d). To study the detailed interaction between miR-5590-3p and SNHG14, we mutated the predicted miR-5590-3p site on SNHG14 for luciferase reporter assay (Fig. 2e). The overexpression of miR-5590-3p decreased the luciferase activity of SNHG14 WT, but failed to affect the luciferase activity of SNHG14 Mut, confirming that miR-5590-3p interacted with SNHG14 at the predicted binding site (Fig. 2e). The correlation curve depicted the negative correlation between miR-5590-3p and SNHG14 expressions in DLBCL samples (Fig. 2f). Moreover, we detected the reciprocal regulation between miR-5590-3p and SNHG14 in DLBCL cells. RT-qPCR analysis confirmed the increase of miR-5590-3p level under the transfection of miR-5590-3p mimics in DLBCL cells (Fig. 2g). SNHG14 was downregulated by miR-5590-3p mimic transfection in DLBCL cells (Fig. 2h). In turn, downregulation of SNHG14 by shRNAs led to increased level of miR-5590-3p in DLBCL cells (Fig. 2i). Together, SNHG14 interacted with and inhibited miR-5590-3p in DLBCL.

### SNHG14/miR-5590-3p induced interaction of DLBCL cells with CD8+ T cells and triggered apoptosis of CD8+ T cells through PD-1/PD-L1 immune checkpoint

**Fig. 3:**
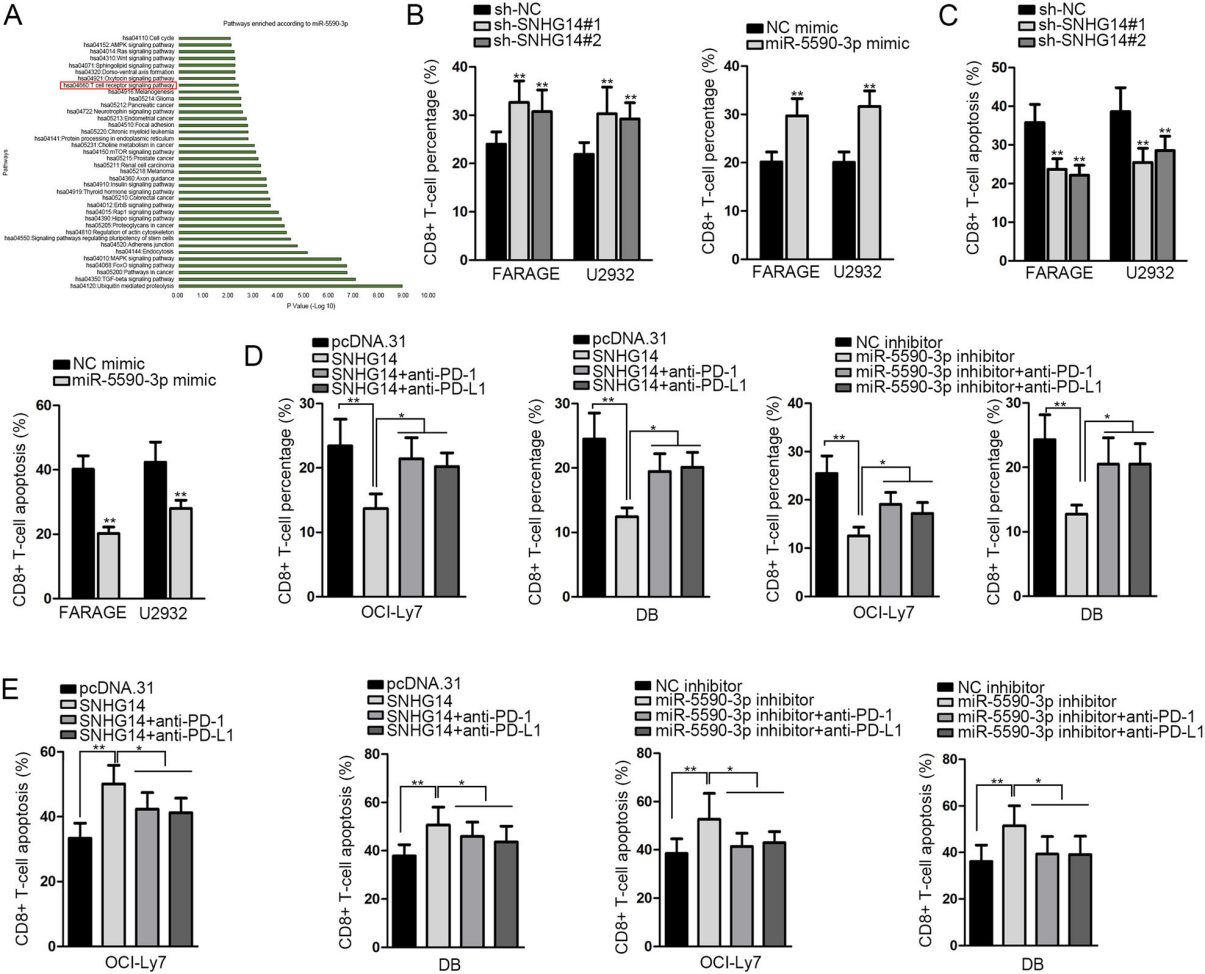
SNHG14/miR-5590-3p altered the activity of CD8+ T cells. **a** Target genes for miR-5590-3p were obtained from Starbase3.0. KEGG analysis of miR-5590-3p pathways based on these genes was carried out by DAVID6.8. The miR-5590-3p related pathways (*P* < 0.01) were presented. **b**–**c** CD8+ T cells were co-cultured with FARAGE and U2932 cells with SNHG14 silence or miR-5590-3p overexpression. CD8+ T cell percentage and apoptosis were analyzed by flow cytometry analysis. **d**–**e** CD8+ T cells were co-cultured with OCI-Ly7 and DB cells with SNHG14 overexpression or miR-5590-3p silence. The co-culture system was treated with or without anti-PD-1 or anti-PD-L1. CD8+ T cell percentage and apoptosis were analyzed by flow cytometry analysis. *P < 0.05, **P < 0.01

Thereafter, we explored the downstream mechanism of SNHG14/miR-5590-3p. We obtained the putative target genes for miR-5590-3p from Starbase3.0, and carried out KEGG pathway analysis through online bioinformatics tool DAVID6.8 (https://david.ncifcrf.gov/home.jsp). It was shown that miR-5590-3p was significantly related to 58 pathways, among which T cell receptor signaling pathway caught our attention (Fig. 3a). As reported, tumor cells interacted with and disturbed the cytotoxic functions of CD8+ T cells in tumor microenvironment^6–8^. Therefore, we deduced that SNHG14/miR-5590-3p could alter CD8+T cells in DLBCL. To mimic the tumor microenvironment, the DLBCL cells were co-cultured with CD8+ T cells. Flow cytometry analysis revealed that percentage of CD8+ T cells increased and apoptosis of CD8+ T cells decreased under the knockdown of SNHG14 or overexpression of miR-5590-3p in FARAGE and U2932 cells (Fig. 3b, c). Furthermore, since PD-1/PD-L1 interaction could inhibit the activity of CD8+ T cells and enhance the immune evasion in tumors^11–13^, we deduced that SNHG14/miR-5590-3p could regulate the alteration of CD8+ T cells through PD-1/PD-L1 checkpoint. To confirm our hypothesis, we applied antibodies against PD-1 and PD-L1 to block PD-1/PD-L1 in the co-culture system. As a result, overexpression of SNHG14 or knockdown of miR-5590-3p in OCI-Ly7 and DB cells reduced the ratio of CD8+ T cells and induced the apoptosis of CD8+ T cells, and such phenomenon could be reversed by the treatment of anti-PD-1 or anti-PD-L1 (Fig. 3d, e). Together, SNHG14/miR-5590-3p induced interaction of DLBCL cells with CD8+ T cells and triggered apoptosis of CD8+ T cells through PD-1/PD-L1 immune checkpoint.

### SNHG14/miR-5590-3p regulated ZEB1/PD-L1 in DLBCL cells

**Fig. 4:**
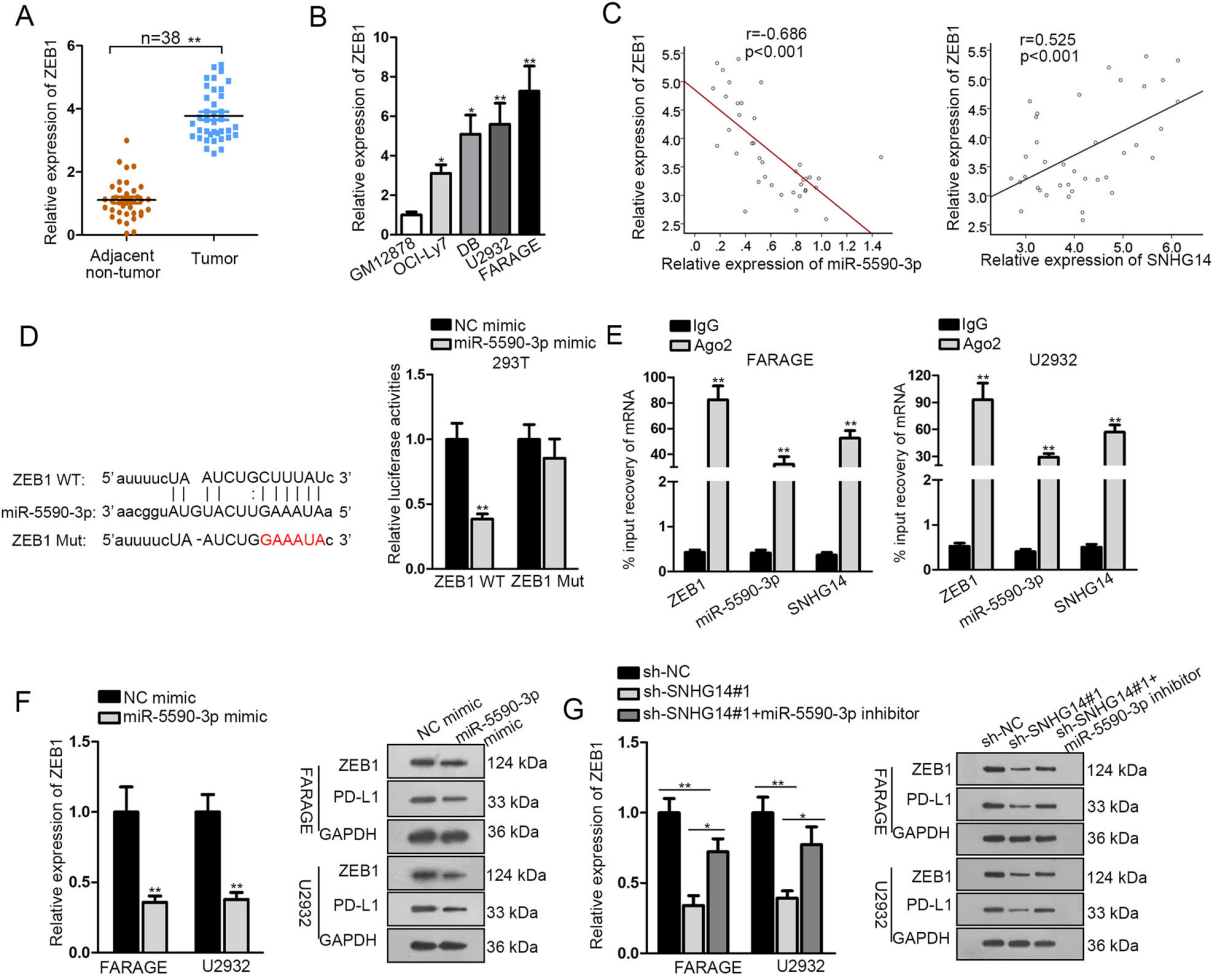
SNHG14 upregulated ZEB1 through miR-5590-3p to activate PD-L1. **a** Expression of ZEB1 in DLBCL tissues versus paired para-tumorous tissues was detected by RT-qPCR. **b** Expression of ZEB1 in DLBCL cell lines and normal cell line was detected by RT-qPCR. **c** Pearson’s correlation curve showed that ZEB1 was positively correlated with SNHG14 and negatively correlated with miR-5590-3p in DLBCL tissues. **d** Interacting sequences on ZEB1 for miR-5590-3p were obtained from Starbase3.0 and mutated by altering them with the complementary sequences. Luciferase reporter assay was performed to detect the interaction between ZEB1 with miR-5590-3p. **e** RT-qPCR analysis following the RIP assay was conducted to confirm the interaction between miR-5590-3p with ZEB1 and SNHG14 in DLBCL cells. **f** Expressions of ZEB1 mRNA and protein and PD-L1 protein upon miR-5590-3p overexpression in DLBCL cells were detected by RT-qPCR and western blot. **g** Expressions of ZEB1 mRNA and protein and PD-L1 protein upon indicated transfection in DLBCL cells were detected by RT-qPCR and western blot. *P < 0.05, **P < 0.01

Further, we asked how SNHG14/miR-5590-3p regulated PD-1/PD-L1 immune checkpoint. Interestingly, we found that ZEB1 was a potential target for miR-5590-3p. Several studies have revealed that ZEB1 could upregulate the expression of PD-L1 to contribute to the immune evasion in cancer cells^28,29^. Therefore, we deduced that SNHG14/miR-5590-3p regulated ZEB1 in DLBCL to activate PD-1/PD-L1. First, we confirmed that ZEB1 was upregulated in DLBCL tissues and cell lines (Fig. 4a, b). Pearson’s correlation analysis depicted that ZEB1 was negatively related to miR-5590-3p and positively related to SNHG14 in DLBCL samples (Fig. 4c). Then, the interacting sequences on ZEB1 for miR-5590-3p and the mutant sequences were presented in Fig. 4d. Forced expression of miR-5590-3p decreased the luciferase activity of ZEB1 WT instead of ZEB1 Mut (Fig. 4d). RIP analysis followed by RT-qPCR assay demonstrated that ZEB1 mRNA, miR-5590-3p, and SNHG14 were all immunoprecipitated by Ago2 (Fig. 4e). Additionally, overexpression of miR-5590-3p reduced the mRNA and protein levels of ZEB1, as well as the protein level of PD-L1 in DLBCL cells (Fig. 4f). Silence of SNHG14 reduced the mRNA expression of ZEB1 and the protein expressions of ZEB1 and PD-L1 in DLBCL cells, and such effect was countervailed by the transfection of miR-5590-3p inhibitor (Fig. 4g). In collection, SNHG14 induced ZEB1/PD-L1 via miR-5590-3p in DLBCL cells.

### ZEB1 transcriptionally upregulated PD-L1 and SNHG14 in DLBCL

**Fig. 5:**
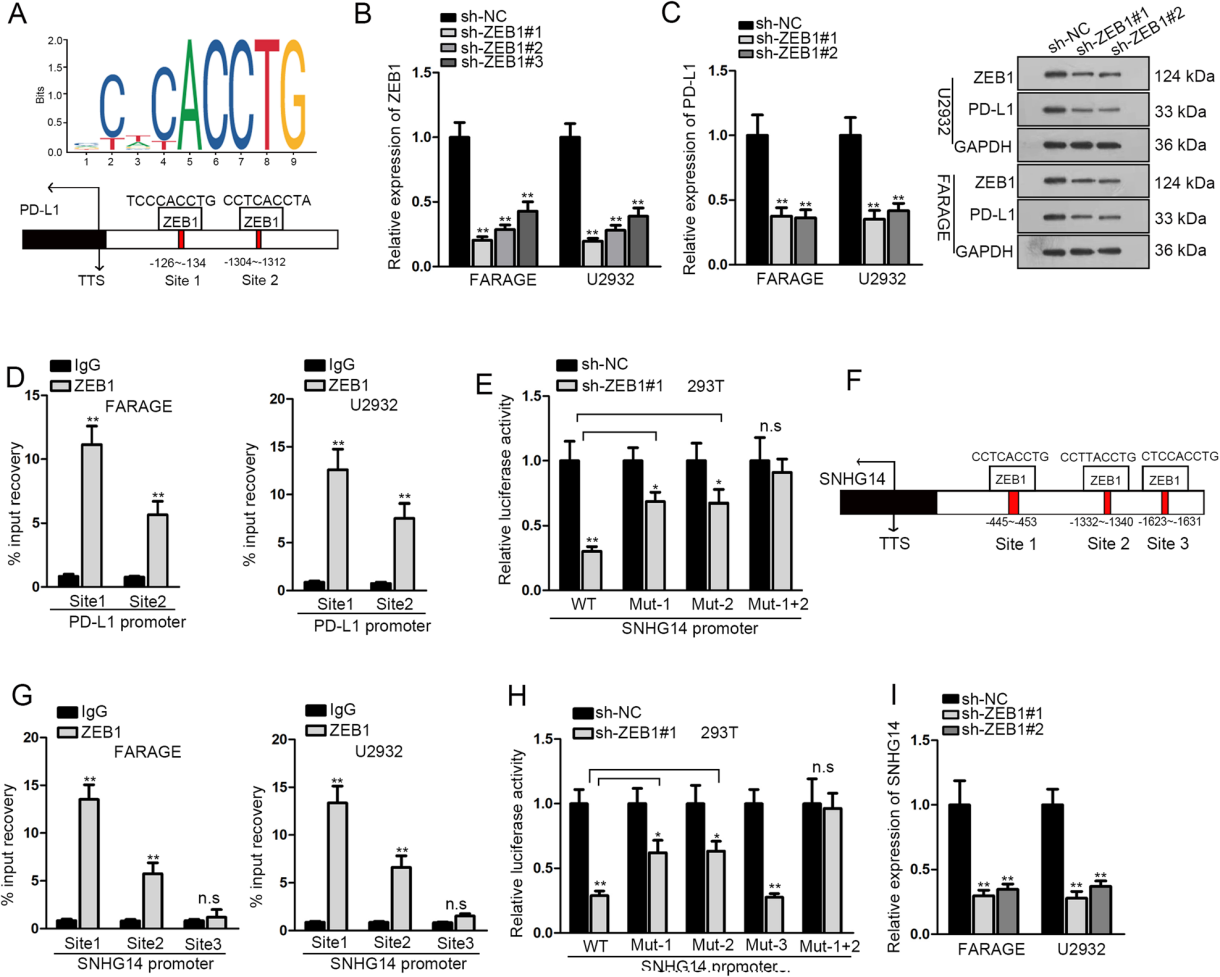
ZEB1 transcriptionally activated PD-L1 and SNHG14. **a** DNA motif of ZEB1 and the predicted binding sites on PD-L1 promoter were obtained from JASPAR. **b** Silence of ZEB1 in FARAGE and U2932 cells was confirmed by RT-qPCR. **c** Expressions of PD-L1 mRNA and protein and ZEB1 protein upon ZEB1 silence in DLBCL cells were detected by RT-qPCR and western blot. **d**–**e** ChIP and luciferase reporter assays showed that ZEB1 bound to both sites 1 and 2 on PD-L1 promoter. **f** The predicted ZEB1 binding sites on SNHG14 promoter were obtained from JASPAR. **g**–**h** ChIP and luciferase reporter assays showed that ZEB1 bound to both sites 1 and 2, rather than site 3 on SNHG14 promoter. **i** Expression of SNHG14 upon ZEB1 silence in DLBCL cells was detected by RT-qPCR. *P < 0.05, **P < 0.01

Subsequently, we investigated the detailed regulation of ZEB1 on PD-L1. ZEB1 is a well-known transcription factor which could regulate the transactivation of target genes^23,24^. Therefore, we tried to detect whether ZEB1 regulated PD-L1 transcriptionally in DLBCL. Through applying the JASPAR tool (http://jaspar.genereg.net/), we identified 2 binding sites on PD-L1 promoter for ZEB1. The DNA motif of ZEB1 and the predicted ZEB1 sites on PD-L1 promoter were presented in Fig. 5a. We confirmed the silence of ZEB1 in FARAGE and U2932 cells by RT-qPCR and western blot (Fig. 5b, c). The mRNA and protein levels of PD-L1 were decreased by ZEB1 knockdown in two DLBCL cell lines (Fig. 5c). ChIP assay depicted that the DNA fragments of PD-L1 promoter containing either site 1 or site 2 were enriched in the precipitates of ZEB1 antibody (Fig. 5d). Luciferase activity of PD-L1 promoter reporter was attenuated by ZEB1 silence, and such attenuation could be partly reversed by mutating site 1 or site 2, however, ZEB1 silence failed to alter the luciferase activity of PD-L1 promoter reporter when mutating both sites 1 and 2 (Fig. 5e). These data suggested that both site 1 and site 2 were responsible for the binding of ZEB1 on PD-L1 promoter.

In addition, accumulating studies revealed that TFs could regulate the transactivation of lncRNAs^55,56^. Therefore, we wondered whether ZEB1 was also responsible for the upregulation of SNHG14 in DLBCL. Interestingly, we found through JASPAR that SNHG14 promoter contained 3 potential binding sites for ZEB1 (Fig. 5f). ChIP analysis demonstrated that only SNHG14 promoter with site 1 and site 2 could be immunoprecipitated by ZEB1 antibody (Fig. 5g). The luciferase activity of wild type SNHG14 promoter reporter was weakened by ZEB1 silence, and respective mutation of site 1 or site 2, rather than site 3, could partially restore the luciferase activity, and mutation of both sites 1 and 2 could fully recover the luciferase activity (Fig. 5h). The downregulation of SNHG14 expression in DLBCL cells by ZEB1 knockdown was confirmed as well (Fig. 5i). Jointly, it was validated that ZEB1 transcriptionally upregulated PD-L1 and SNHG14 in DLBCL.

### SNHG14 repressed the activity of CD8+ T cells and progression of DLBCL cells through ZEB1

**Fig. 6:**
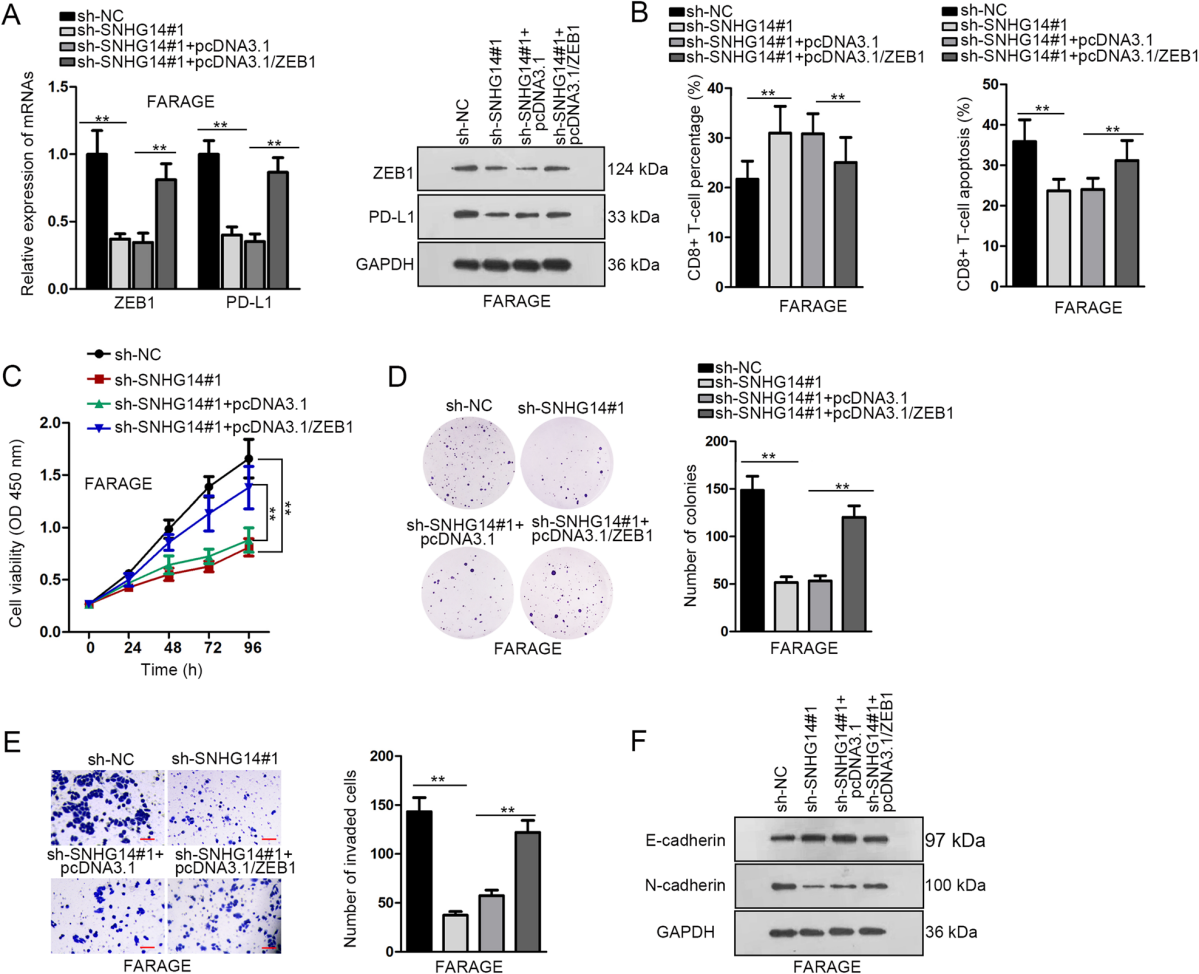
SNHG14 inhibited CD8+ T cell activity and promoted DLBCL progression through ZEB1. FARAGE cells were transfected with sh-NC, sh-SNHG14#1, sh-SNHG14#1+pcDNA3.1, or sh-SNHG14#1+pcDNA3.1/ZEB1. **a** The mRNA and protein expressions of ZEB1 and PD-L1 in FARAGE cells were detected by RT-qPCR and western blot analysis. **b** CD8+ T cells were co-cultured with transfected FARAGE cells. Percentage and apoptosis of CD8+ T cells were analyzed by flow cytometry assay. **c**–**d** Proliferation of FARAGE cells under indicated transfection was detected by CCK-8 and colony formation assay. **e** Invasion of FARAGE cells under indicated transfection was detected by transwell invasion assays. Scale bar: 100 μm. **f** Expression of E-cadherin and N-cadherin in FARAGE cells under indicated transfection was detected by western blot analysis. **P < 0.01

To figure out whether ZEB1 was required for the regulation of SNHG14 on the immune evasion and progression of DLBCL cells, we conducted rescue assays in FARAGE cells. First, we confirmed that ZEB1 and PD-L1 mRNA and protein expressions were reduced by SNHG14 depletion and recovered by the overexpression of ZEB1 (Fig. 6a). In the co-culture system, silence of SNHG14 in FARAGE cells increased the ratio of CD8+ T cells and decreased the apoptosis of CD8+ T cells, and such effect could be abrogated by the co-transfection of pcDNA3.1/ZEB1 (Fig. 6b). Silencing SNHG14 inhibited the proliferation and invasion of DLBCL cells, and forced expression of ZEB1 abolished the inhibitory effects of SNHG14 silence (Fig. 6c, e). Also, the increased E-cadherin level and decreased N-cadherin level under SNHG14 knockdown in FARAGE cells were reversed by the overexpression of ZEB1 (Fig. 6f). In a word, these findings suggested that SNHG14 promoted the immune evasion and progression of DLBCL cells through ZEB1.

### SNHG14 aggravated tumor growth of DLBCL through PD-1/PD-L1 immune checkpoint in vivo

**Fig. 7:**
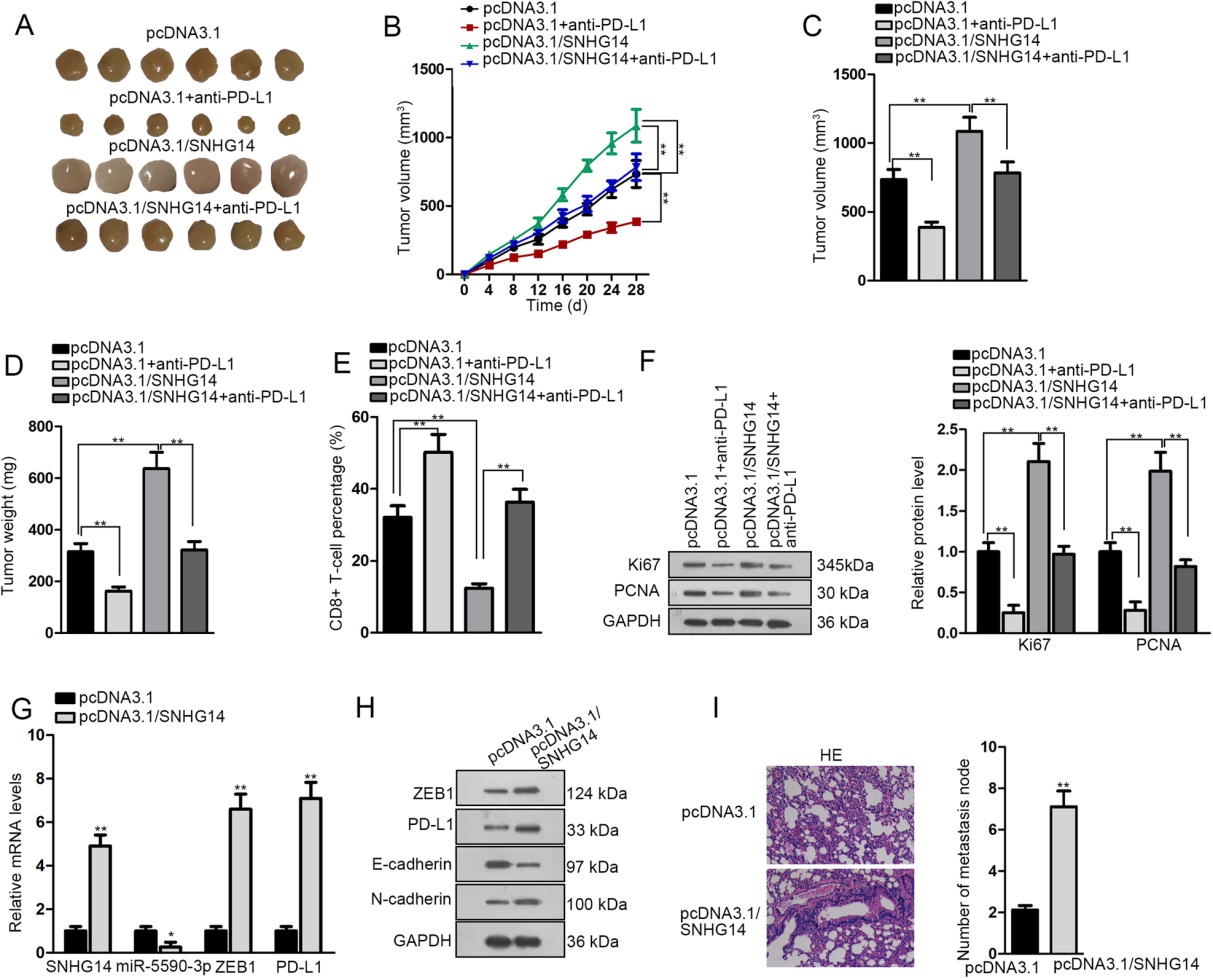
SNHG14 promoted DLBCL tumor growth and immune evasion in vivo. A20 cells were transfected with pcDNA3.1 or pcDNA3.1/SNHG14 and injected into the mice. The mice were treated with or without anti-PD-L1. **a** Pictures of xenografts in mice of each group. **b** Growth curve of tumor generation in mice of each group. **c**–**d** Quantification of volume and weight of tumors in mice of each group after 28 days of injection. **e** Percentage of CD8+ T cells in xenografts of mice of each group was detected by flow cytometry. **f** Western blot results of Ki67 and PCNA expressions in the tumors generated by mice of each group. **g**–**h** Expressions of SNHG14, miR-5590-3p, ZEB1, and PD-L1 in mice under the overexpression of SNHG14 was detected RT-qPCR and western blot. **i** HE staining showed the metastasis nodes in mice under the overexpression of SNHG14 compared with control mice. Scale bar: 100 μm. *P < 0.05, **P < 0.01

Finally, the effect of SNHG14 on DLBCL tumorigenesis was detected through animal experiments. A20 cells were transfected with pcDNA3.1 or pcDNA3.1/SNHG14. Then the transfected cells were injected into nude mice treated with or without anti-PD-L1to establish the xenografts. We observed that the overexpression of SNHG14 led to the generation of bigger tumors, whereas the use of anti-PD-L1 in mice led to the generation of smaller tumors. Also, the application of PD-L1 antibody abrogated the inductive effect of SNHG14 on tumor growth in mice (Fig. 7a). The overexpression of SNHG14 facilitated, whereas the use of anti-PD-L1 retarded the DLBCL tumor growth in mice, and anti-PD-L1 abrogated the facilitative effect of SNHG14 overexpression on tumor growth (Fig. 7b). The tumor volume and weight were increased by SNHG14 overexpression whereas were decreased by anti-PD-L1, and anti-PD-L1 reversed the increase of tumor volume and weight caused by SNHG14 overexpression (Fig. 7c, d). In addition, the overexpression of SNHG14 reduced the ratio of CD8+ T cells, whereas the use of anti-PD-L1 increased the ratio of CD8+ T cells and also impaired the reductive effect of SNHG14 overexpression on CD8+ T cell ratio (Fig. 7e). Also, levels of proliferation markers (Ki67 and PCNA) in tumors were increased by SNHG14 overexpression, whereas decreased by anti-PD-L1, and PD-L1 antibody reversed the inductive impact of SNHG14 overexpression on the level of proliferation markers (Fig. 7f). Besides, we confirmed that tumors generated by the injection of A20 cells transfected with pcDNA3.1/SNHG14 presented higher expressions of SNHG14, ZEB1, and PD-L1 and lower expression of miR-5590-3p than control (Fig. 7g, h). Also, the level of E-cadherin decreased, whereas level of N-cadherin increased upon SNHG14 overexpression in vivo (Fig. 7h). According to the HE staining assay, metastatic nodules of DLBCL were increased responding to the overexpression of SNHG14 in vivo (Fig. 7i). To be concluded, SNHG14 aggravated tumor growth of DLBCL through PD-1/PD-L1 immune checkpoint in vivo.

## Discussions

More and more reports have suggested lncRNAs as essential therapeutic and diagnosis biomarkers in the carcinogenesis of human cancers during past decades. A number of studies have stated that lncRNAs elicit regulatory functions in DLBCL as well. For example, SMAD5-AS1 inhibited proliferation of DLBCL through Wnt/β-catenin pathway^37^; HOTAIR was upregulated in DLBCL and predicted unsatisfactory prognosis of DLBCL patients^36^. Herein, we discovered through microarray and bioinformatics analysis that SNHG14 was upregulated in DLBCL samples, indicating the participation of SNHG14 in DLBCL. Previously, SNHG14 was suggested by several works to pose carcinogenic impact on diverse types of cancers, such as gastric cancer, clear cell renal cell carcinoma, and breast cancer^38–40^. This study was the first to establish the relation between SNHG14 and DLBCL. Loss-of-function assays revealed that SNHG14 exerted oncogenic impacts by positively affecting proliferation, invasion, and EMT in DLBCL cells.

Mechanistically, we revealed the action of SNHG14 as a miRNA sponge in DLBCL. As reported by former works, lncRNAs could interfere with the miRNA-mRNA interaction through sequestering miRNAs so as to relieve mRNAs from miRNA-caused repression of protein translation^44,45^. Also, lncRNA-mediated ceRNA mechanism has been demonstrated in DLBCL^37^. Our findings showed that among the candidate miRNAs targeted by SNHG14, miR-5590-3p was overtly downregulated in DLBCL samples and cell lines, and presented strong affinity with SNHG14, indicating that SNHG14 functioned in DLBCL mainly through miR-5590-3p. Expectedly, we firstly confirmed the interaction between SNHG14 and miR-5590-3p and the reciprocal inhibition between them.

Furthermore, we conducted KEGG analysis through bioinformatics tool and found that miR-5590-3p target genes were significantly enriched in T cell receptor signaling pathway. It has been recognized that tumor cells could alter the T cell activity to escape from the anti-tumor immune response, which contributed to the survival of tumor cells^4,5^. Notably, previous literatures pointed out that tumor cells could interact with and induce the apoptosis of the CD8+ T cells to promote tumor growth and metastasis^57,58^. In current study, we firstly revealed that inhibiting SNHG14 or inducing miR-5590-3p could attenuate the activity of CD8+ T cells and induce the apoptosis of CD8+ T cells. Moreover, mounting studies have provided compelling evidence that neutralizing PD-1 or PD-L1 to block the PD-1/PD-L1 could activate CD8+ T cells and abrogate the immune evasion of tumor cells^11–13^, suggesting that antibody against PD-1 and PD-L1 is an effective clinical immune therapy for cancers^14–17^. The application of anti-PD-1 and anti-PD-L1 for the immunotherapy in lymphoma has been studied as well^18,19^. Our findings firstly showed that SNHG14/miR-5590-3p altered CD8+ T cell activity through PD-1/PD-L1 axis, suggesting that targeting SNHG14 potentially improved the efficacy of immunotherapy in DLBCL through PD-1/PD-L1.

Further, we firstly found that ZEB1 was a target for miR-5590-3p. Formerly, several works argued that ZEB1 could upregulate the expression of PD-L1 in cancer cells and contribute to the inactivation of CD8+ T cells^28,29^. Besides, the tumor-promoting impact of ZEB1 on DLBCL has been validated by a former study as well^27^. These findings prompted us to deduce that ZEB1 mediated the regulation of SNHG14 on the immune evasion and progression of DLBCL cells. Unsurprisingly, we confirmed the interaction between ZEB1 and miR-5590-3p and suggested that SNHG14 induced ZEB1 expression to activate PD-L1 through sponging miR-5590-3p. In addition, we firstly demonstrated in detail that ZEB1 bound to the promoter of PD-L1 to activate its transcription. More interestingly, we found that ZEB1 could also target the promoter of SNHG14 and regulate the transactivation of SNHG14, suggesting that SNHG14/miR-5590-3p/ZEB1 formed a positive regulatory feedback loop in DLBCL cells. Accordingly, previous literatures have revealed that ZEB1 could activate the transcription of certain lncRNAs in tumor cells^59,60^, but the effect of ZEB1 on SNHG14 was firstly revealed in this study. Finally, rescue assays indicated that ZEB1 was required for the regulation of SNHG14 on the alteration of CD8+ T cell activity and progression of DLBCL cells. Through in animal experiments, we suggested that SNHG14 promoted tumor growth of DLBCL through regulating the PD-1/PD-L1 immune checkpoint.

## Conclusions

**Fig. 8:**
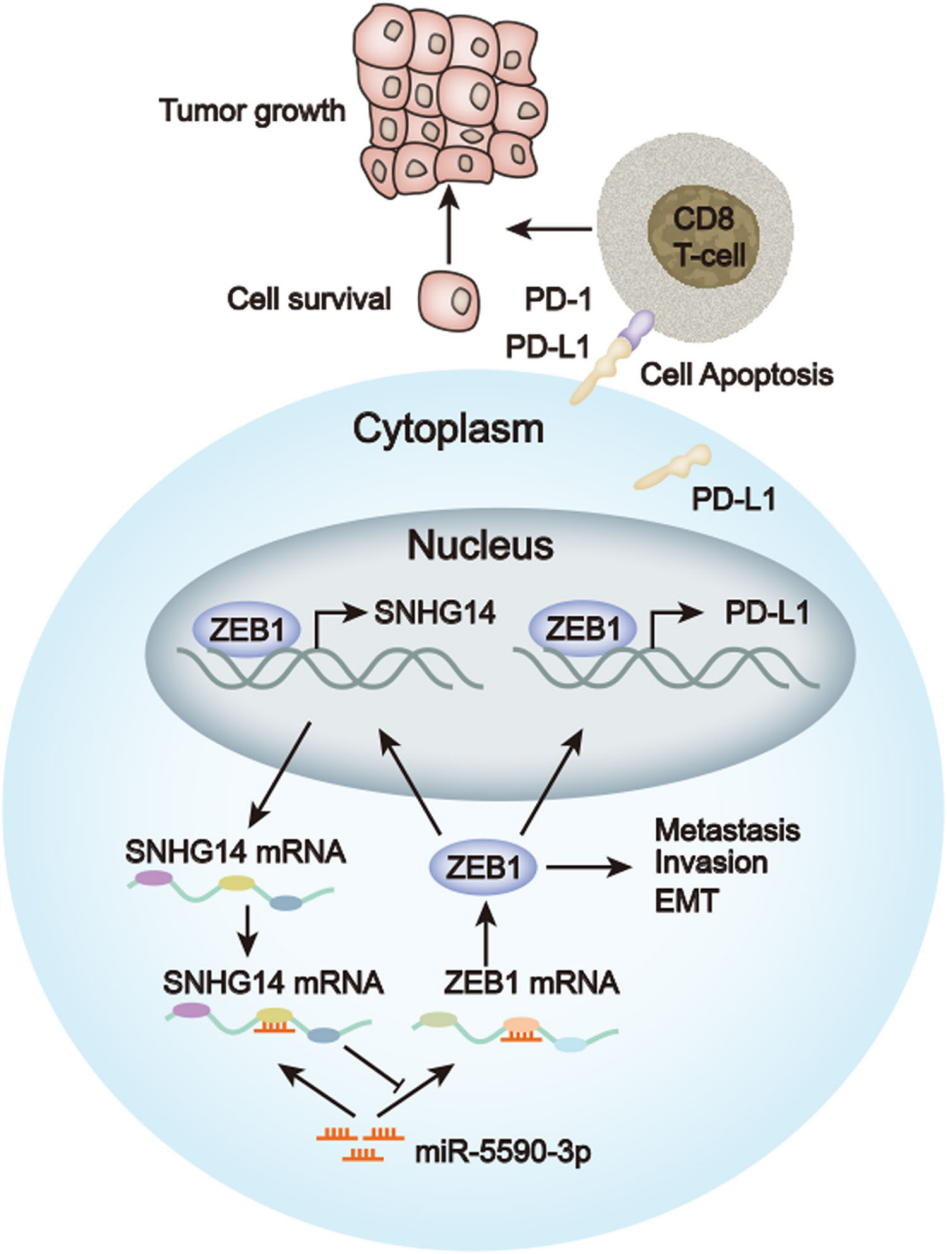
LncRNA SNHG14/miR-5590-3p/ZEB1 positive feedback loop promoted diffuse large B-cell lymphoma progression and immune evasion through regulating PD-1/PD-L1 checkpoint

Our study firstly uncovered that SNHG14 promoted proliferation, invasion, EMT and tumor growth of DLBCL in vitro and in vivo. Mechanistically, we elucidated that SNHG14/miR-5590-3p/ZEB formed a positive feedback loop in DLBCL cells to activate PD-L1, leading to the inactivation of CD8+ T cells and contributing to the immune evasion of DLBCL cells (Fig. 8). These findings further indicated that SNHG14 could be a promising target for the immunotherapy through PD-L1/PD-1 blockade in DLBCL.
